# UnCorrupt SMILES: a novel approach to de novo design

**DOI:** 10.1186/s13321-023-00696-x

**Published:** 2023-02-14

**Authors:** Linde Schoenmaker, Olivier J. M. Béquignon, Willem Jespers, Gerard J. P. van Westen

**Affiliations:** Computational Drug Discovery, Drug Discovery and Safety, Leiden Academic Centre for Drug Research, Einsteinweg 55, Leiden, The Netherlands

**Keywords:** SMILES correction, Invalid SMILES, Molecular transformer, De novo drug design, Analog generation

## Abstract

Generative deep learning models have emerged as a powerful approach for de novo drug design as they aid researchers in finding new molecules with desired properties. Despite continuous improvements in the field, a subset of the outputs that sequence-based de novo generators produce cannot be progressed due to errors. Here, we propose to fix these invalid outputs post hoc. In similar tasks, transformer models from the field of natural language processing have been shown to be very effective. Therefore, here this type of model was trained to translate invalid Simplified Molecular-Input Line-Entry System (SMILES) into valid representations. The performance of this SMILES corrector was evaluated on four representative methods of de novo generation: a recurrent neural network (RNN), a target-directed RNN, a generative adversarial network (GAN), and a variational autoencoder (VAE). This study has found that the percentage of invalid outputs from these specific generative models ranges between 4 and 89%, with different models having different error-type distributions. Post hoc correction of SMILES was shown to increase model validity. The SMILES corrector trained with one error per input alters 60–90% of invalid generator outputs and fixes 35–80% of them. However, a higher error detection and performance was obtained for transformer models trained with multiple errors per input. In this case, the best model was able to correct 60–95% of invalid generator outputs. Further analysis showed that these fixed molecules are comparable to the correct molecules from the de novo generators based on novelty and similarity. Additionally, the SMILES corrector can be used to expand the amount of interesting new molecules within the targeted chemical space. Introducing different errors into existing molecules yields novel analogs with a uniqueness of 39% and a novelty of approximately 20%. The results of this research demonstrate that SMILES correction is a viable post hoc extension and can enhance the search for better drug candidates.

**Graphical Abstract:**

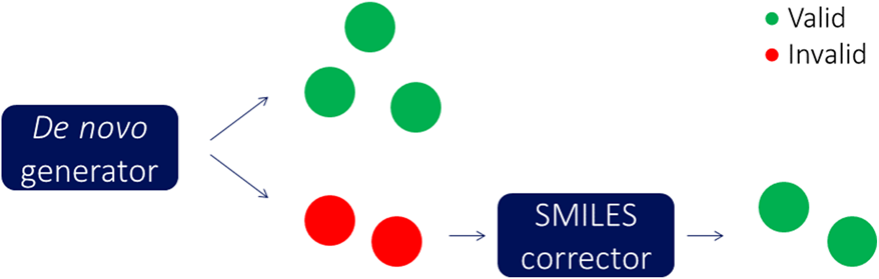

**Supplementary Information:**

The online version contains supplementary material available at 10.1186/s13321-023-00696-x.

## Introduction

Finding molecules with desired characteristics is a key aspect of drug discovery [1]. Advances in the field have led to a variety of approaches for discovering promising compounds [2]. Specifically, methods like enumerated virtual libraries and de novo drug design are gaining attention, as they expand the search into a larger chemical space than existing physical compound libraries. This enhances the probability of finding drug candidates with specific desired properties and increases chemical diversity.

Over the past decade, a variety of generative models for de novo drug design have been published [3]. These models are trained to generate molecules based on a training set and are sampled to create new molecules. When reinforcement learning is incorporated into generative models, they can also be used to generate compounds that fulfill specific objectives. For the majority of de novo generators, molecules are represented by the Simplified Molecular-Input Line-Entry System (SMILES) [4, 5]. This is the case because SMILES and other molecular line notations are linear and therefore compatible with well-established generative models from the field of natural language processing (NLP) [6]. Previous research has shown the applicability of recurrent neural networks (RNNs), autoencoders, generative adversarial networks (GANs), and other generative models [7, 8].

A disadvantage of using SMILES and similar molecular line notations is that the generated sequences can be invalid. A valid SMILES sequence needs to adhere to specific syntax rules and also be chemically correct. The validity of different generative models has been compared in the GuacaMol benchmark, a standardized evaluation framework for generative models [9]. This showed that a general RNN-based model has a percentage of invalid outputs of around 4%. For generative autoencoders, the invalid rate was higher with around 15% invalid SMILES. With regards to generative variational autoencoders (VAE), the validity of outputs has also been reported to vary more, exemplifying the difficulty of sampling a continuous space [10]. The main disadvantage of these invalid outputs is that they cannot be progressed, therefore random samples of the chemical space will be absent or a bias might even be introduced towards molecules that are easier to correctly generate.

Therefore, considerable efforts have been made to increase the validity of generated molecules. To this end, different molecular line representations like DeepSMILES and SELF-referencing Embedded Strings (SELFIES) have been designed, but not widely adopted [11, 12]. In addition to this, graph representations can be used that directly represent molecules as graphs [13]. An advantage of graph-based models is that they almost exclusively generate valid outputs [14]. However, graph-based models are more challenging to apply because they have a higher computational cost and a lower generation speed [15–17]. Another approach that has been used to increase the number of valid outputs of VAEs, is to apply context-free grammar and attribute grammar [18, 19]. However, these approaches have the disadvantage that they reduce the search space. Theoretically, invalid SMILES sequences could also be corrected using translator models as used in the field of grammatical error correction (GEC) [20]. These types of models have an encoder-decoder architecture and can be trained to translate sequences into other sequences. Interestingly, Zheng et al*.* already showed that the principles from this field can be applied to correct syntax mistakes in short SMILES sequences, in the context of molecular building blocks [21]. In other SMILES-based tasks, translator models have also been successfully applied [22–24].

Although extensive research has been carried out on reducing the number of invalid outputs, no previous study has investigated the potential of these incorrect outputs. These outputs could be a useful source for the generation of new molecules and increase generator efficiency. In addition to this, it is probable that errors occur more frequently in more complex or longer sequences [25]. Therefore, the objective of this study is to fix these incorrect sequences and analyze the resulting molecules. Additionally, SMILES correction could offer a new approach to de novo drug design, in which the chemical space around existing molecules is expanded by introducing and fixing errors.

To train the SMILES corrector, a data set with pairs of invalid and valid SMILES was created. The number of errors introduced into the valid SMILES was varied to explore the potential benefits of training with multiple errors. The best-performing SMILES corrector was then used to correct invalid outputs from four de novo generation case studies: a general RNN, a VAE, a GAN and a conditional RNN model. Using chemical similarity and property distributions, the resulting fixed molecules were then compared to the training set and to molecules originally generated by the four de novo models. Lastly, the SMILES corrector was used to correct mistakes introduced into selective Aurora kinase B inhibitors to evaluate if local sequence exploration can be used to expand the nearby chemical space. Taken together, the work presented here provides the first exploration of the potential of invalid molecular representations for de novo drug design.

## Methods

### Data sets and preprocessing

Invalid SMILES and their corresponding valid molecule are required to train the SMILES corrector. However, to the best of our knowledge, no collections of manually corrected pairs like this exist. Therefore, mistakes were introduced into correct SMILES to create a training set of invalid-valid pairs, similarly to previously established techniques in GEC [26–28]. All training sets are based on standardized molecules without specified stereochemistry from the Papyrus data set (version 5.5) [29, 30]. For standardization the ChEMBL structure pipeline was used [31]. Each molecule was standardized, and solvents, salts, and duplicate fragments were removed. After standardization, errors were introduced based on random permutations and the SMILES syntax rules. Additionally, valence errors were introduced by increasing the bond order of bonds connected to atoms with a ‘full’ valence and by adding small fragments from the GDB-8 database to such atoms [32]. A list of all the perturbations used to introduce the errors can be found in Additional file 1: Figure S1). To determine the influence of using input SMILES with multiple errors, sets with different numbers of errors per input (2, 3, 5, 8, 12, and 20) were created. The interval increased as the number of errors to be introduced increased, because the effect of adding additional errors was expected to be smaller. For all sets, sequences containing more than 200 tokens were removed. Afterward, the data sets all contained roughly 1.3 million invalid-valid pairs. The distribution of the errors occurring in these sets, assessed using RDKit, can be found in (Additional flile 1: Figure S5).

The aforementioned Papyrus data set was also used to train the de novo generators. For training DrugEx, the data set was preprocessed as described by Liu et al*.* [33]. To train GENTRL, only SMILES that could be parsed by the tokenizer were included, resulting in a training set of 1.2 million sequences. For ORGANIC, a smaller training set of 15000 diverse molecules was created from the Papyrus data set using RDKit’s sphere exclusion algorithm [34].

For the target-directed case study, a data set was created to train and evaluate the predictor models. To this end, high and medium-quality activity data was collected for human Aurora kinase A (AURKA) (UniProt accession O14965) and human Aurora kinase B (AURKB) (UniProt accession Q96GD4) from the Papyrus data set. The data set for AURKA consisted of 1232 bioactivity data points with pChEMBL values ranging from 3.4 to 11.0. The data set for AURKB consisted of 1131 bioactivity data points with pChEMBL values ranging from 4.1 to 11.0. For the selectivity window model, an additional data set was created with compounds with experimental K_i_ values for both of the targets [35]. This data set consisted of 849 relative affinity values ranging from -1.6 (indicating selectivity towards AURKA) to 3.0 (indicating selectivity towards AURKB).

### Generative models

Real-world sets of incorrect SMILES were used for validation, based on four case studies: a general RNN model, a target-directed RNN model, a VAE, and a GAN.

DrugEx, created by Liu et al*.* was used as the RNN-based generator [33]. The pretrained version of DrugEx was made by training on the standardized Papyrus set using the code available on GitHub [36].

In order to create the target-directed RNN, the pretrained DrugEx model was fine-tuned on the molecules tested on AURKA and/or AURKB, and optimized towards selective AURKB compounds using reinforcement learning. To tune the target-directed RNN and to predict the bioactivity of novel molecules, three prediction models were created; two models that were trained to predict the bioactivity of compounds on AURKA and on AURKB, and one selectivity window model that predicts a compound’s selectivity for AURKB over AURKA. To this end, quantitative structure–activity relationship (QSAR) regression models were constructed using Scikit-learn [37]. The molecules were described using 19 physicochemical properties and extended-connectivity fingerprints of the molecules. Both were calculated with the RDKit and the fingerprints had a radius of 3 bonds and were folded to 2048 bits (ECFP6). Four different machine learning methods were compared: Naïve Bayesian (NB), Random Forest (RF), K-nearest neighbors (KNN), and Support Vector Machine (SVM). For each model hyperparameter optimization was performed using Optuna’s Tree-structured Parzen Estimator algorithm for 64 trials to maximize the Matthews Correlation Coefficient [38].

For the VAE generative model case study, the generator GENTRL was pretrained on the previously described Papyrus set [39, 40]. During training, a batch size of 50 was used for 10 epochs with a learning rate of 10^–4^ similar to the procedure described by Zhavoronkov et al*.*

ORGANIC was used as the GAN and trained on the previously described set of 15,000 diverse molecules from the Papyrus data set [34, 41]. First, generator and discriminator pretraining was done for 240 and 50 epochs, respectively. Afterwards, the model was trained with the objective to adhere to Lipinski’s rule of five for 110 epochs.

After training, the different generators from each case study were used to create 1 million sequences. The valid sequences created by these models will be referred to as the readily generated sequences to distinguish them from valid SMILES corrector outputs.

### Error analysis of SMILES

To gather information on the validity of model outputs, invalid SMILES were identified using RDKit [42]. SMILES that could not be converted into molecules were regarded as invalid. The invalid SMILES were also classified into six categories based on their RDKit error message: syntax error, unclosed ring, parentheses error (extra open parentheses or extra close parentheses), bond already exists (dual occurrence of a bond between the same two atoms), aromaticity error (a combination of non-ring atom marked aromatic and kekulization errors) and valence error (an atom’s maximum number of bonds is exceeded).

### SMILES correction model

To create a model that can correct invalid SMILES, transformer models were constructed using PyTorch [43]. The transformer model used in this research is adapted from Ben Trevett’s *Pytorch Seq2Seq* model [44]. For this model, the input and output sequences were tokenized using TorchText tokenizers and the output sequence was reversed. The SMILES tokenizer was based on the one described by Olivecrona et al*.* and most tokens consisted of single characters, except for tokens denoting atoms with two-letter atom symbols, atom descriptions between brackets, and numbers following the % sign (Additional file 1: Table S1) [45]. In addition to these tokens, start, stop and padding tokens were used, resulting in vocabulary sizes of between 101 and 110 tokens.

The transformer model architecture was based on the model described in the paper by Vaswani et al*.*, but it differed in that learned positional encodings were introduced along with a standard Adam optimizer. Moreover, the implementation did not have label smoothing (Additional file 1: Figure S3) [46]. The learning rate for the optimizer was 0.0005. The encoder took in an embedding layer as well as a positional embedding layer, both with a dimension of 256 and a dropout of 0.1. It contained 3 layers of multi-head attention and position-wise feed-forward mechanisms. The multi-head attention layers had a dimension of 256 and 8 heads. The position-wise feed-forward mechanism had a dimension of 512 and a Rectified Linear Unit (ReLU) activation function. After each of these layers, a dropout of 0.1 was applied after which layer normalization was performed. The encoder generated as many context vectors as there were tokens. The decoder worked similarly to the encoder, but it had two multi-head attention mechanisms: one that used the target as input and another that used the encoder representations. The decoder also had a linear layer before the prediction.

The SMILES corrector models were trained on 90% of the invalid-valid pairs from the synthetic data sets and evaluated on the other 10% of the invalid and valid sequences from the synthetic data set, and 10,000 invalid outputs from each of the generative models. The models were trained for 20 epochs using a batch size of 16. At each epoch, the percentage of valid SMILES and the molecule reconstruction rate on the evaluation set were calculated using RDKit. This reconstruction rate was defined as the fraction of translator outputs that represented the same molecule as their corresponding original target molecule. The model with the highest SMILES validation rate on the evaluation set was saved. For the case studies, the percentage of valid SMILES after SMILES correction was determined as well as the percentage of valid and invalid outputs that were changed compared to their input.

### SMILES corrector for exploration

To determine the potential of using SMILES correction for the expansion of the nearby chemical space, errors were introduced into known ligands using the previously described error introduction method and corrected using the SMILES corrector. As a starting point, compounds from the Papyrus set that are selective for AURKB were used. First, a random error was introduced into the SMILES sequences of these molecules and this was repeated 1000 times, after which duplicate sequences were removed. Finally, the errors were fixed using the best-performing SMILES corrector.

### Comparison of fixed outputs

Evaluation metrics from the GuacaMol distribution-learning benchmarks and MOSES benchmark were used to compare the readily generated and fixed molecules to the training set [9, 47]. The uniqueness of generated molecules was defined as the fraction of unique canonicalized SMILES in a sample of 10,000 valid sequences. The novelty was determined by the percentage of molecules in a set of 10,000 generated, valid molecules that were not present in a set of 100,000 reference molecules. In order to assess the similarity, the similarity to nearest neighbor (SNN), fragment similarity, and scaffold similarity were determined following the same approach as in the MOSES benchmark [47]. The SNN was calculated by taking the average of the Tanimoto similarity between 1024 bit ECFP4 fingerprints of the generated molecules and their most similar neighbor molecules from the reference set. For the fragment similarity, the frequency distribution of BRICS fragments was compared. For the scaffold similarity, the same was done based on the Bemis-Murcko scaffolds. For all the similarity metrics 10,000 generated molecules were compared to reference sets of 100,000 molecules. Lastly, the Kullback − Leibler (KL) divergence of the physicochemical property distributions of generated molecules and the reference set was calculated for the following properties: Bert’s complexity index (BertzCT), octanol–water partition coefficient (*log*P), molecular weight (MW), topological polar surface area (TPSA), number of hydrogen bond donors and acceptors, number of rotatable bonds, number of aliphatic and aromatic rings and the fraction of sp^3^ hybridized carbons (CSP3) [48]. The distribution of the continuous descriptors was represented by a smoothed histogram to allow for fast comparison of large sets (Additional file 1: Figure S6). The KL divergences (D_KL_) of the different properties (i) were aggregated into one score following the method from GuacaMol using;

$$\mathrm{score} = \frac{1}{k}\sum_{i}^{k}\mathrm{exp}({-D}_{KL,i})$$

### Docking in AURKB

Novel compounds generated by SMILES corrector exploration of selective AURKB ligands were docked in the crystal structure of AURKB (Protein Data Bank identifier: 4AF3). This structure was prepared for docking with ICM Pro (version 3.9-2d) (Molsoft LLC, San Diego, CA) by removing water molecules, and adding and optimizing hydrogen atoms [49]. The binding site was defined using the default settings to select the residues surrounding the co-crystalized ligand (VX-680), and default parameters were used for docking. The final results were visualized using PyMOL v2.5.2. [50].

## Results and discussion

### Prevalence and type of errors

To gain insights into the frequently occurring errors, 1 million sequences were generated and invalid output sequences were analyzed. For the pretrained and target-directed RNN models, 5.7 and 4.7% of the generated sequences were invalid, respectively. The percentage of invalid sequences was slightly higher for the GAN (9.5%), whereas the VAE had the highest percentage of invalid outputs (88.9%). A very similar validity of RNN-based models was also found in previous benchmarks [9, 47]. Specific results on the RNN-based model that was used (DrugEx) have also shown that fine-tuning and reinforcement learning slightly increase validity [33]. The validity of the GAN that was used is consistent with the one reported in the MOSES benchmark [47]. However, it should be noted that the validity of ORGANIC has been found to be highly variable depending on the training set [34]. The lower validity of VAE outputs is consistent with other studies and is likely caused by regions in the latent space that are far removed from the training data [10, 14, 51].

**Fig. 1:**
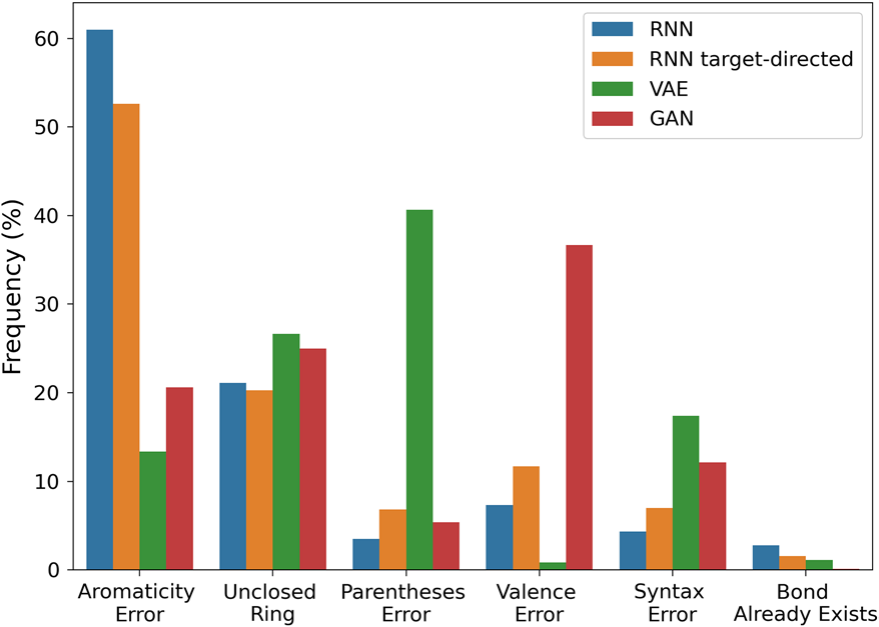
Distribution of the types of errors that occur in the invalid sequences generated by the de novo generators. Duplicate sequences were deleted and the remaining erroneous sequences were categorized based on the error messages from RDKit

To better understand the invalid SMILES the parsing errors captured by the RDKit were classified into six different error types (Fig. 1). It should be noted that in the case of sequences with multiple types of errors only the first error is reported and the chemical validity of sequences with grammar errors cannot be assessed. Interestingly, while the RNN-based models and GAN mainly produced chemistry-related errors, the VAE outputs contain more SMILES grammar errors. Qualitative analysis of invalid SMILES produced by a similar VAE has previously shown that this type of generator often has problems matching pairs of parenthesis and ring symbols [52]. Regarding the chemistry-related errors, aromaticity mistakes were the most prevalent for the RNN-based generators whereas valence errors were most prevalent for the GAN models. This lower prevalence of other errors suggests that RNN and GAN models are better able to learn the SMILES grammar. De novo generators that evade the problem of SMILES validity by using SELFIES or graph representations have also been developed, but are currently less widely used than SMILES-based generators [12, 14, 53, 54]. Overall, these results demonstrate that the prevalence and nature of invalid outputs of different SMILES-based generative models vary and that a variety of different errors have to be rectified in order to correct them.

### Performance of the SMILES corrector

**Fig. 2:**
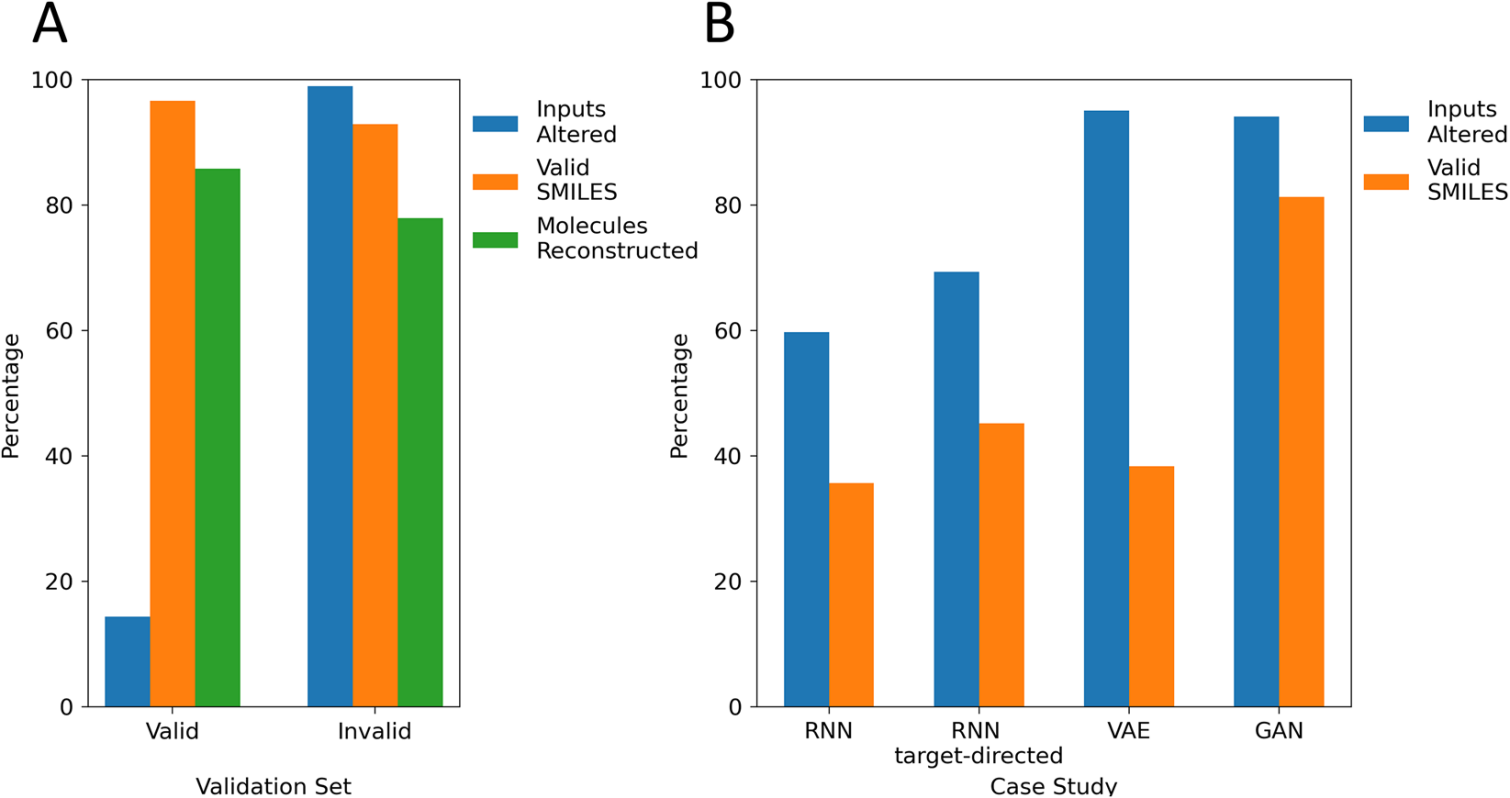
Performance of the SMILES corrector on different sets colored by the metrics used. The performance is given as the percentage of inputs that are different from their respective outputs (blue), valid outputs (orange), and outputs that are identical to their target molecules (green). The corrector was evaluated on valid and invalid SMILES from the evaluation set (**A**) and invalid SMILES from the four case studies (**B**)

The SMILES corrector was trained on the task of fixing invalid SMILES. It was evaluated on an evaluation test set containing both synthetic errors with their corresponding valid SMILES, and invalid outputs generated by the de novo generators (Fig. 2A). Evaluation using the synthetic test set showed that 93% of these invalid SMILES could be fixed. The molecule reconstruction rate of the translator was 78%. This rate is in line with the findings of Bjerrum et al*.* which showed that the reconstruction rate is lower for translation between differently represented molecules. This indicates that fixed molecules do not necessarily represent the original molecule but that in most cases the intended molecule will be generated [24].

To test for overcorrection, valid SMILES were used to evaluate the corrector. This showed that the percentage of valid sequences that were altered during translation was low (14%), indicating that mainly erroneous parts of SMILES were being changed. Interestingly, this percentage is very similar to transformer overcorrection when applied to spelling correction [55]. Based on this it can be concluded that the corrector can distinguish between correct and incorrect sequences.

The SMILES corrector had a lower performance when applied to invalid SMILES from the generative models exemplified by the percentage of valid outputs ranging from 35 to 80%, with the errors from the GAN being the easiest to correct (Fig. 2B). This validity rate range is similar to the results reported by Zheng et al*.*, in the related task of correcting invalid SMILES grammar in reactants [21]. Previous research in GEC has highlighted that performance is associated with the model’s error detection and error correction ability [56]. They concluded that their transformer model was able to generalize error detection to a real-world evaluation whereas correction is more difficult. In our case, the drop in performance could be partly explained by insufficient error detection illustrated by the lower percentage of inputs that had been altered by the translator. Interestingly, for the VAE the percentage of altered inputs was 90% but the validation rate was still low, indicating that finding the right correction is challenging for the model. In general, the relatively high percentage of unaltered sequences and the low validity rate indicate that more representative training pairs are necessary for training the SMILES corrector.

### Training with multiple errors per input improves performance

**Fig. 3:**
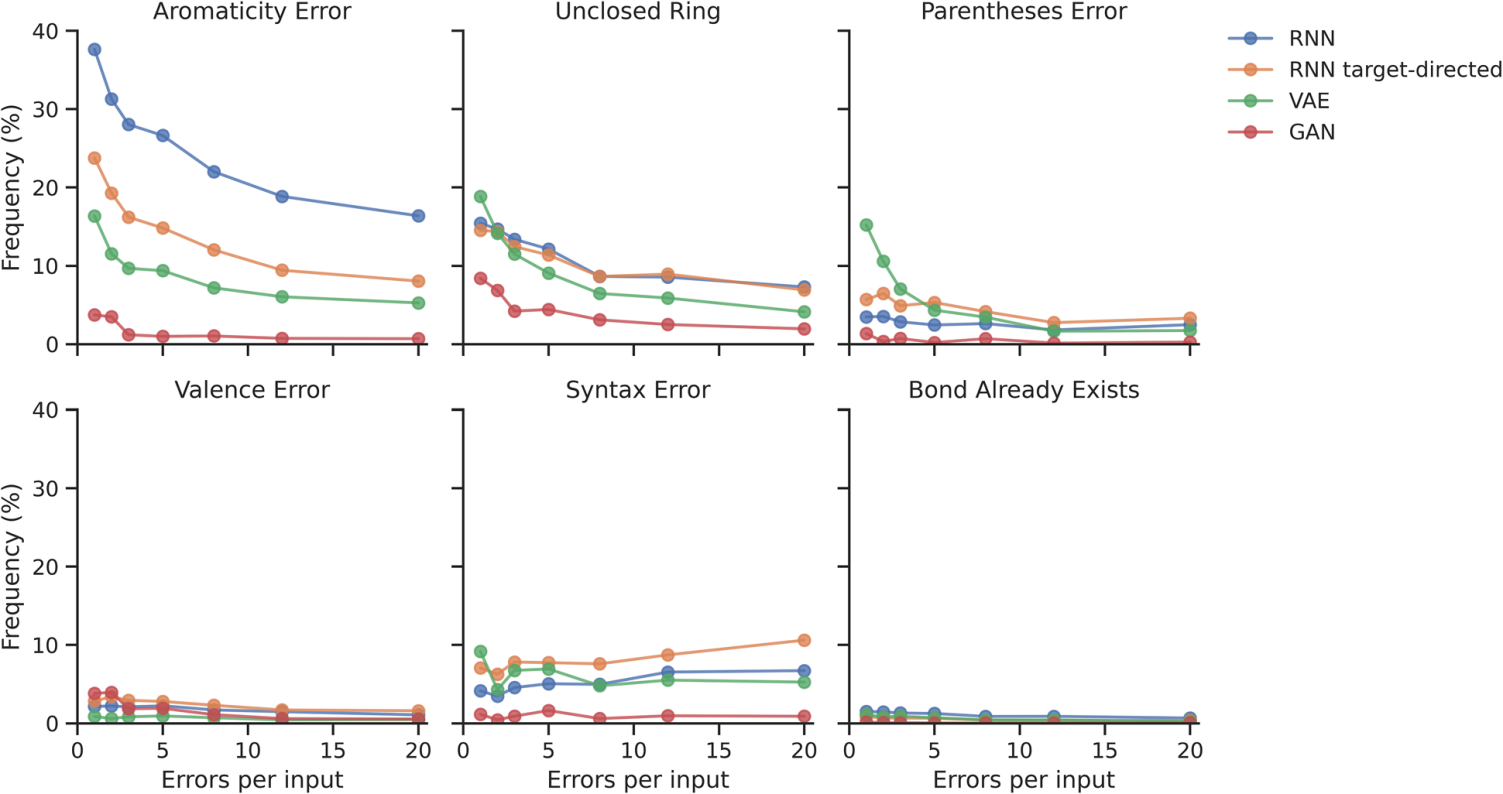
Errors in the outputs of SMILES corrector models trained with multiple errors per input sequence. SMILES corrector models were trained with a different number of errors per input sequence and evaluated on invalid SMILES from the four case studies. Results are given as the percentage of a specific error in the SMILES corrector outputs

To assess the effect of training transformer models to correct sequences with multiple errors, different models were evaluated using invalid outputs from the four de novo generators (Fig. 3). For the baseline corrector model multiple types of errors remained in the output sequences. Increasing the number of errors the SMILES corrector was trained on, mainly decreased the number of remaining aromaticity errors and unclosed rings. For the VAE outputs, there was also a decrease in parentheses errors. However, there was a slight increase in the number of remaining syntax errors compared to the baseline corrector model when the number of mistakes increased beyond two per input, irrespective of the generator type.

Overall, the validity increased when models were trained with inputs containing more errors, but training with more than 12 errors per input did not increase the validity much further, with on average 80% validity for 20 errors per input compared to 78% for 12 errors per input. The reconstruction rate of the latter model is 70.5% on the validation set with 1 error per input. This model achieved a validity of 62, 68, 80, and 95% when used on outputs from the RNN, targeted RNN, VAE, and GAN generators, respectively. In addition to this, more than 86% of input sequences were altered for all case studies. These findings suggest some generators produce more invalid SMILES that are difficult to correct. This means that developing generator-specific correction models, based on invalid generator outputs, could be an interesting topic for future research. The beneficial effect of training with multiple errors is consistent with earlier studies on error correction which found that a model implements more corrections when it has been trained to distrust the inputs more [57]. An additional explanation for this might be that a subsection of the sequences from the case studies contained interacting errors that were difficult to correct for the baseline model, as has also been found for GEC previously [58, 59].

### Effect of fixing SMILES

To establish whether SMILES correction could yield novel, complementary molecules, the fixed molecules were analyzed and compared to readily generated molecules from the three general generative models and to the training set (Table. 1). 97% of the molecules generated by the SMILES corrector were unique. Interestingly, more than 97% of the molecules were novel compared to the readily generated molecules and the average SNN between the two sets was relatively low (0.45). This indicates that SMILES correction was able to generate compounds that were novel and dissimilar compared to the ones that had already been generated.

**Table 1:** Comparison of fixed, generated, and training set molecules

Case – reference	Uniqueness^a^	Novelty	Similarity			KL divergence
			SNN	Fragment	Scaffold	
Fixed—generated	0.97 ± 0.05	0.97 ± 0.06	0.45 ± 0.09	0.93 ± 0.10	0.49 ± 0.31	0.77 ± 0.15
Fixed—train		1.00 ± 0.00	0.41 ± 0.02	0.92 ± 0.12	0.30 ± 0.06	0.80 ± 0.23
Generated—train	1.00 ± 0.00	1.00 ± 0.00	0.39 ± 0.10	0.88 ± 0.18	0.40 ± 0.17	0.75 ± 0.28

The mean ± standard deviation for the 3 general generative model case studies is given. Uniqueness is the fraction of unique molecules in a sample of 10,000 valid molecules. Novelty is the fraction of 10,000 molecules that are not present in a sample of 100,000 from the reference set. For the similarity metrics, 10,000 molecules were compared to 100,000 molecules from the reference set. SNN is the similarity to the nearest neighbor. Fragment and scaffold similarity are calculated by comparing the frequency distribution of different fragments or scaffolds compared to the reference set. KL divergence describes the similarity of the physicochemical property distributions of 10,000 molecules compared to the reference set

^a^Uniqueness is calculated for the fixed and generated set

The readily generated and fixed molecules did not differ in terms of their novelty and similarity compared to the training set. Additionally, both the fixed molecules and readily generated ones approximated the training set to a similar extent regarding their property distributions. These findings suggest that SMILES correction generates molecules that are as useful as the molecules created by the generator that the errors originated from.

### Local sequence exploration

**Fig. 4:**
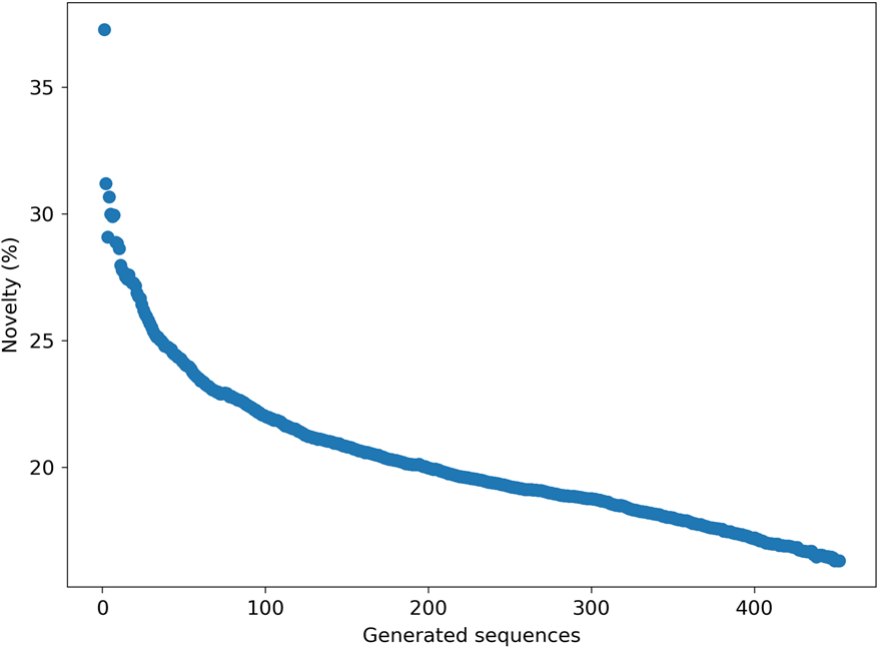
Novelty of analog exploration using the SMILES corrector for different numbers of outputs. Novelty is given as the average novelty for fixed inputs based on 370 different selective Aurora kinase B ligands

As it was hypothesized that error correction could also be used for the expansion of the nearby chemical space, synthetic errors were introduced and fixed for a set of compounds that were selective for AURKB over AURKA. 97% of output sequences resulting from this local sequence exploration were valid, but they did have a low uniqueness (39%) and novelty (between 16 and 37%) compared to e.g. fragment-based analog generators [60, 61]. This was because in most cases the original molecule was regenerated. In a recent study, Creanza et al*.* created a SMILES-based analog generator that works by sampling SMILES with substitutions [62]. When using five substitutions, this approach has a lower validity (11%) but higher uniqueness (60%) compared to SMILES correction for exploration. On average the novelty when one altered version of an input sequence was corrected was around 37% (Fig. 4). When more than 400 different random erroneous versions were created based on the same sequence the novelty decreased to around 16%. These results, therefore, show that this method of sequence exploration can be used to generate novel compounds, which will be explored further in the next section.

### Applicability on Aurora kinases

**Fig. 5:**
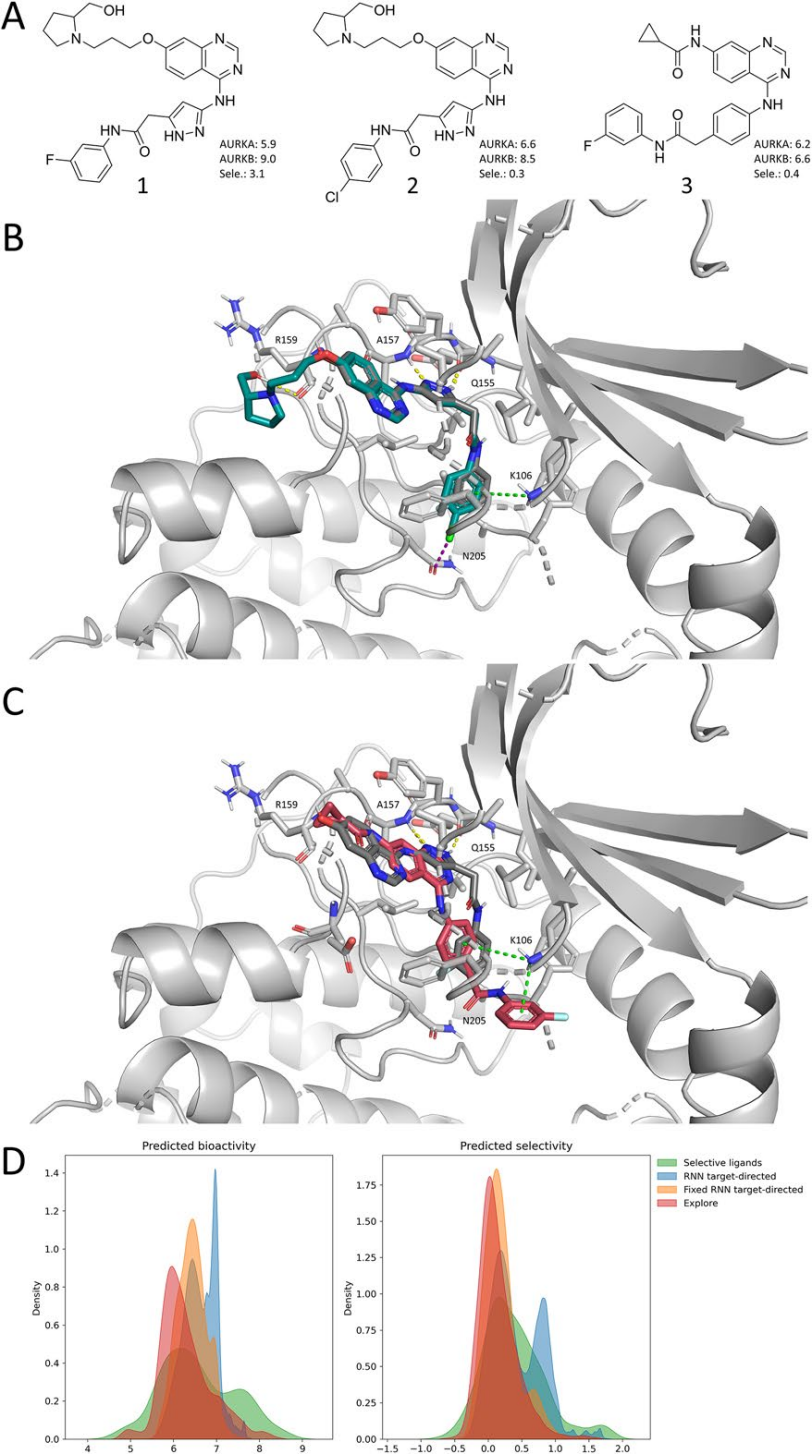
SMILES exploration on Aurora kinases. A an example of novel compounds (2 and 3) generated by the SMILES corrector based on original compound 1, with their measured/predicted bioactivity on Aurora kinase A and B and selectivity of Aurora kinase B over A. B compound 2 (blue) docked into Aurora kinase B (PDB 4AF3) with co-crystalized ligand VX-680 (grey). C compound 3 (pink) docked into Aurora kinase B (PDB 4AF3) with co-crystalized ligand VX-680 (grey). Hydrogen, halogen and pi-cation bonds are displayed as yellow, purple and green dotted lines, respectively. Figures created with Pymol v2.5.2. D distribution of predicted bioactivity (left) and predicted selectivity (right) of molecules generated by RNN target-directed, fixed RNN target-directed, and SMILES exploration compared to the predicted values of the existing ligands with experimentally determined selectivity

To compare SMILES-based exploration to an established method for target-directed de novo generation, its applicability for creating new selective AURKB ligands was tested (Table 2). Compared to the target-directed RNN the molecules resulting from SMILES-based exploration had a higher average SNN to the original compounds. This similarity is comparable to the similarity of the SMILES-based analog generator created by Creanza et al*.* [62]. The distribution of scaffolds generated by SMILES-based exploration was also more similar to the known ligands. The same trend was reflected by the KL divergence score, indicating that SMILES exploration more closely followed the property distributions of the target data set. A few of the novel compounds generated based on an original molecule are illustrated in Fig. 5A. These findings suggest that SMILES exploration can contribute to de novo generation efforts in which the goal is to design compounds that are more similar to existing ones.

**Table 2:** Properties of target-directed RNN and SMILES exploration outputs

	Similarity			KL divergence
	SNN	Fragment	Scaffold	
RNN target-directed	0.38	0.95	0.07	0.64
Fixed RNN target-directed	0.32	0.97	0.05	0.46
Explore	0.85	0.99	0.63	0.81

SNN is the similarity to the nearest neighbor of 10,000 molecules from the generated sets compared to the set of 1627 known AURKA and AURKB ligands. Fragment and scaffold similarity are calculated by comparing the frequency distribution of different fragments or scaffolds compared to the known ligands. KL divergence describes the similarity of the physicochemical property distributions of 10,000 molecules from the generated sets compared to the know ligands

The potential of exploration of the nearby space was also evaluated in terms of the predicted bioactivity and selectivity of the novel compounds generated this way (Fig. 5A–D). Novel compound 2 has similar predicted bioactivities compared to starting compound 1 (Fig. 5A). Interestingly, novel compound 3 has a lower predicted affinity for AURKB but a slightly higher predicted selectivity. Docking of both compounds in AURKB (Protein Data Bank identifier: **4AF3**) shows that they occupy the same region as the co-crystalized ligand (VX-680) with additional stabilizing interactions (Fig. 5B, C). SMILES exploration resulted in compounds with similar bioactivities to already known compounds, but with a higher density for bioactivities of around 6.0 log units, whilst the target-directed RNN was able to produce more compounds with higher predicted activities (Fig. 5D). However, previous studies on derivatization design have suggested that there are also merits to staying closer to known actives, as in the case of Makara et al*.* this does lead to a higher hit rate compared to a target-directed VAE [63].

Together, these results demonstrate that SMILES exploration is suitable for the generation of novel compounds that are similar to a starting compound, although it is less efficient for generating compounds with desirable bioactivities compared to reinforcement learning.

## Conclusions

This project is the first comprehensive investigation of the applicability of deep learning methods to correct invalid sequences in de novo drug design. Commonly used SMILES-based generators produce some percentage of invalid outputs and here it was shown that invalid SMILES are not useless or something to avoid. This study has found that a transformer network trained on synthetic mistakes can fix more than 60% of invalid SMILES originating from different molecular generators with different distributions of error types. SMILES correctors trained on input sequences with multiple errors demonstrated a higher performance. This study also showed that the pretrained SMILES corrector generates novel molecules that follow the same distribution as the original generators and/or set of molecules, hence demonstrating that the SMILES corrector can independently be used for the exploration of the nearby space of molecules of interest.

## Supplementary Information

**Additional file1: Figure S1.** Description of alterations performed to generate the synthetic errors. **Figure S2.** Two examples of molecules with synthetic valence errors. **Figure S3.** Figure of transformer architecture, adapted from a work by Yuening Jia from under CC BY-SA 3.0. **Figure S4.** The errors that are present in the synthetic datasets based on the papyrus dataset. **Figure S5.** The errors that are present in the synthetic datasets based on the papyrus dataset. **Figure S6.** Effect of number of bins on KL divergence score. The plots show the divergence score of smoothened histograms with different numbers of bins compared to the divergence score based on kernel density estimation. **Figure S7.** Performance of QSAR models for Aurora kinase A. **Figure S8.** Performance of QSAR models for Aurora kinase B. **Figure S9.** Performance of selectivity window QSAR models. **Table S1.** Regular expression used for tokenizing the molecular representations. **Table S2.** Comparison of fixed, generated, and training set molecules for the 3 general generative model case studies is given. Uniqueness is the fraction of unique molecules in a sample of 10,000 valid molecules. Novelty is the fraction of 10,000 molecules that are not present in a sample of 100,000 from the reference set. For the similarity metrics, 10,000 molecules were compared to 100,000 molecules from the reference set. SNN is the similarity to the nearest neighbor. Fragment and scaffold similarity are calculated by comparing the frequency distribution of different fragments or scaffolds compared to the reference set. KL divergence describes the similarity of the physiochemical property distributions of 10,000 molecules compared to the reference set.

## Abbreviations

AURKA: Aurora kinase A
AURKB: Aurora kinase B
BertzCT: Bert’s complexity index
CSP3: Sp^3^ hybridized carbons
ECFP: Extended-connectivity fingerprints
GAN: Generative adversarial networks
GEC: Grammatical error correction
GENTRL: Generative tensorial reinforcement learning
KL divergence: Kullback − Leibler divergence
KNN: K-nearest neighbors
LogP: Octanol–water partition coefficient
MW: Molecular weight
NB: Naïve Bayesian
NLP: Natural language processing
QSAR: Quantitative structure–activity relationship
ReLU: Rectified linear unit
RF: Random forest
RNN: Recurrent neural network
SELFIES: SELF-referencing embedded strings
SMILES: Simplified molecular-input line-entry system
SNN: Similarity to nearest neighbor
SVM: Support vector machine
TPSA: Topological polar surface area
VAE: Variational autoencoder

## Declarations

### Availability of data and materials

The data and code required to recreate the results of this paper are available in the following GitHub repository: https://github.com/LindeSchoenmaker/SMILES-corrector.
