# Supplementary material for: UnCorrupt SMILES: a novel approach to de novo design
Source: J Cheminform. 2023 Feb 14;15:22. doi: 10.1186/s13321-023-00696-x (PMC9926805; doi:10.1186/s13321-023-00696-x)
Supplement: Supplementary file 1 — Additional file1: Figure S1. Description of alterations performed to generate the synthetic errors. Figure S2. Two examples of molecules with synthetic valence errors. Figure S3. Figure of transformer architecture, adapted from a work by Yuening Jia from under CC BY-SA 3.0. Figure S4. The errors that are present in the synthetic datasets based on the papyrus dataset. Figure S5. The errors that are present in the synthetic datasets based on the papyrus dataset. Figure S6. Effect of number of bins on KL divergence score. The plots show the divergence score of smoothened histograms with different numbers of bins compared to the divergence score based on kernel density estimation. Figure S7. Performance of QSAR models for Aurora kinase A. Figure S8. Performance of QSAR models for Aurora kinase B. Figure S9. Performance of selectivity window QSAR models. Table S1. Regular expression used for tokenizing the molecular representations. Table S2. Comparison of fixed, generated, and training set molecules for the 3 general generative model case studies is given. Uniqueness is the fraction of unique molecules in a sample of 10,000 valid molecules. Novelty is the fraction of 10,000 molecules that are not present in a sample of 100,000 from the reference set. For the similarity metrics, 10,000 molecules were compared to 100,000 molecules from the reference set. SNN is the similarity to the nearest neighbor. Fragment and scaffold similarity are calculated by comparing the frequency distribution of different fragments or scaffolds compared to the reference set. KL divergence describes the similarity of the physiochemical property distributions of 10,000 molecules compared to the reference set. [file 13321_2023_696_MOESM1_ESM.docx]

Supporting information: UnCorrupt SMILES: a novel approach to *de novo* design

Linde Schoenmaker, Olivier J.M. Béquignon, Willem Jespers & Gerard J.P. van Westen

Regular expressions used for tokenizing the valid and invalid SMILES

Additional file 1: Table S1. Regular expression used for tokenizing the molecular representations.

|  | **Regular expression** |
| --- | --- |
| SMILES | r"(\[[^\]]+]\|Br?\|Cl?\|N\|O\|S\|P\|F\|I\|b\|c\|n\|o\|s\|p\|\(\|\)\|\.\|=\|#\|-\|\+\|\\\\|\\|\/\|:\|~\|@\|\?\|>\|\*\|\$\|\%[0-9]{2}\|[0-9])" |

Types of synthetic errors


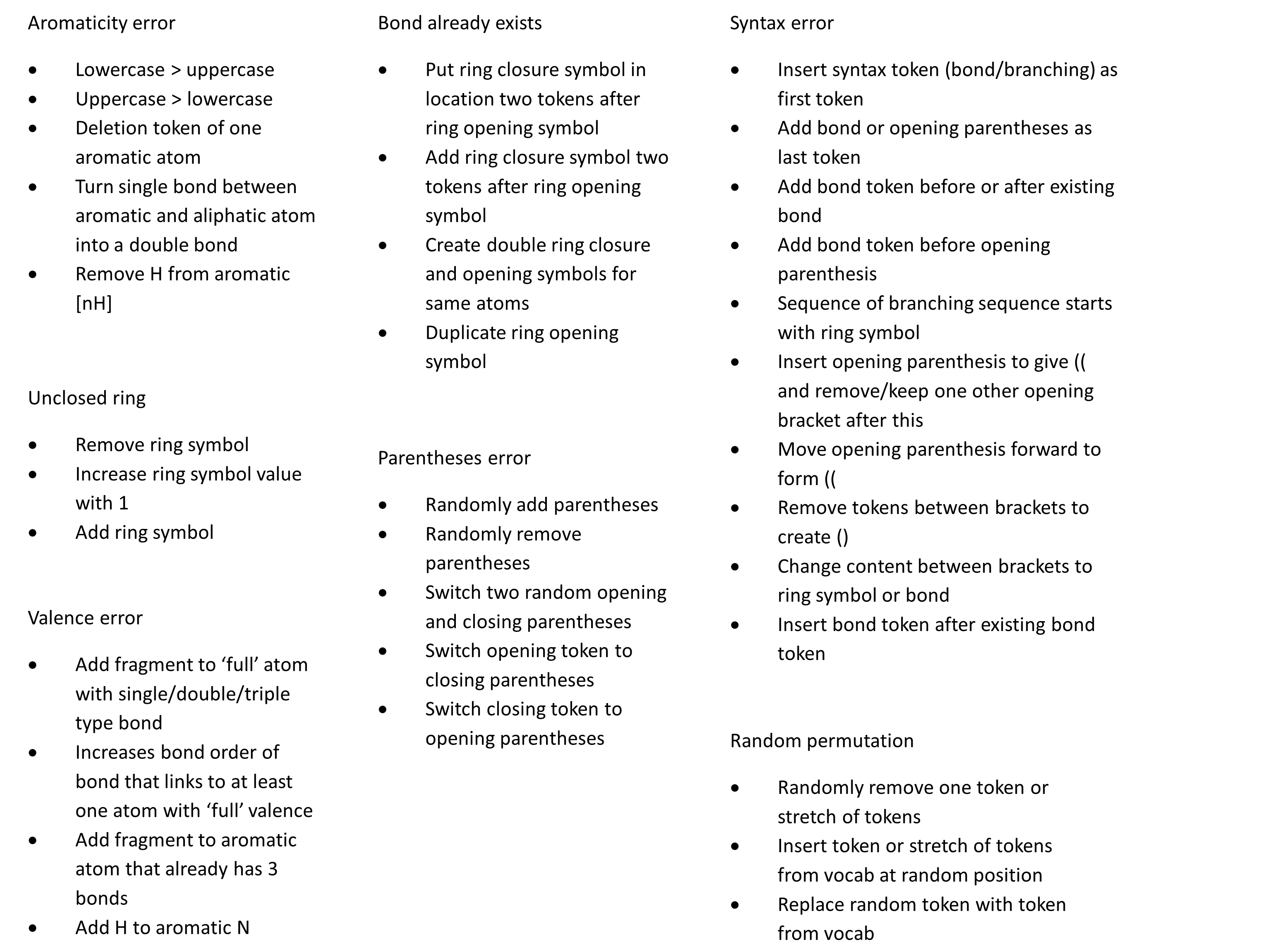


Additional file 1: Figure S1. Description of alterations performed to generate the synthetic errors


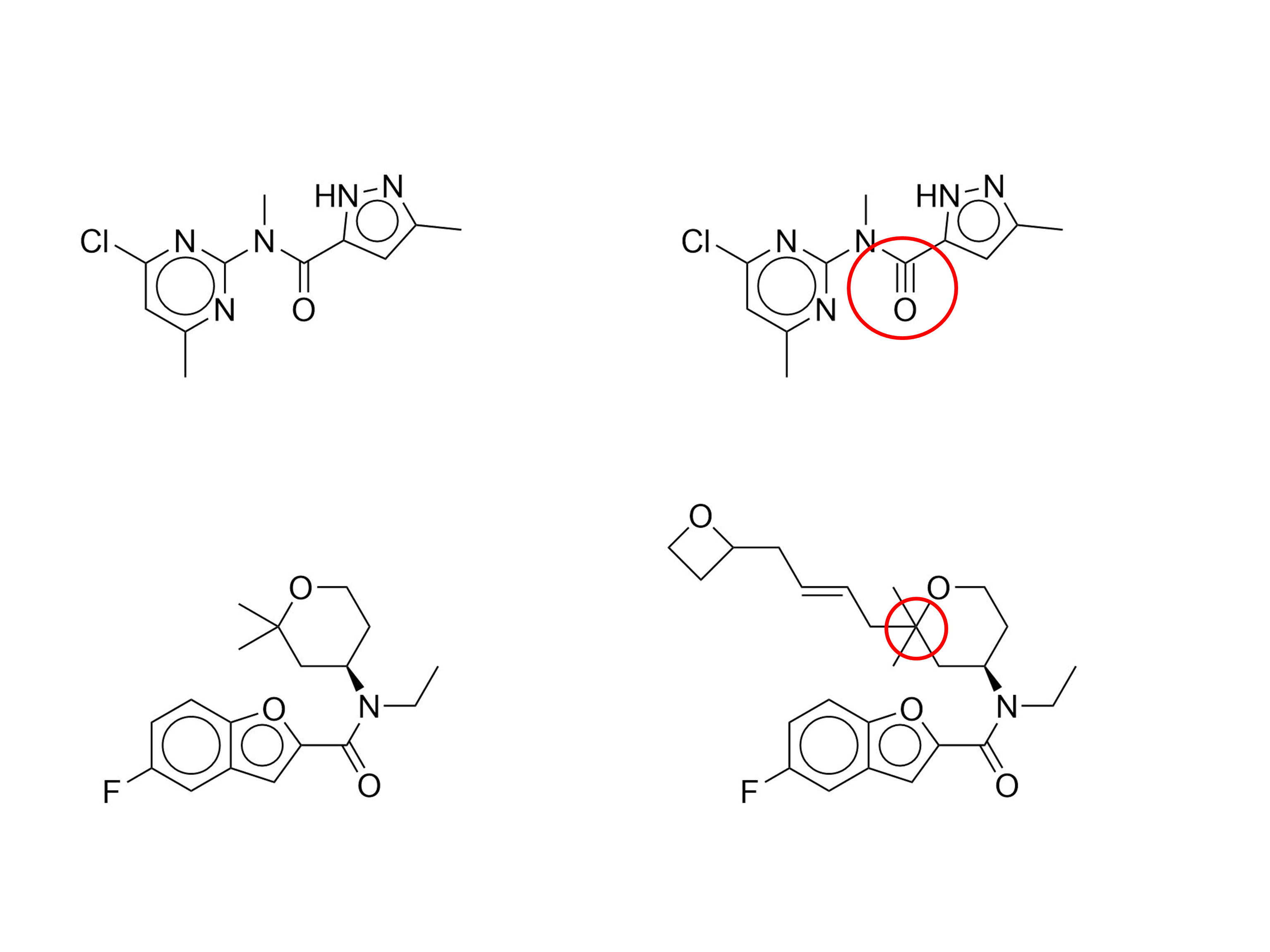


Additional file 1: Figure S2. Two examples of molecules with synthetic valence errors.

Transformer architecture


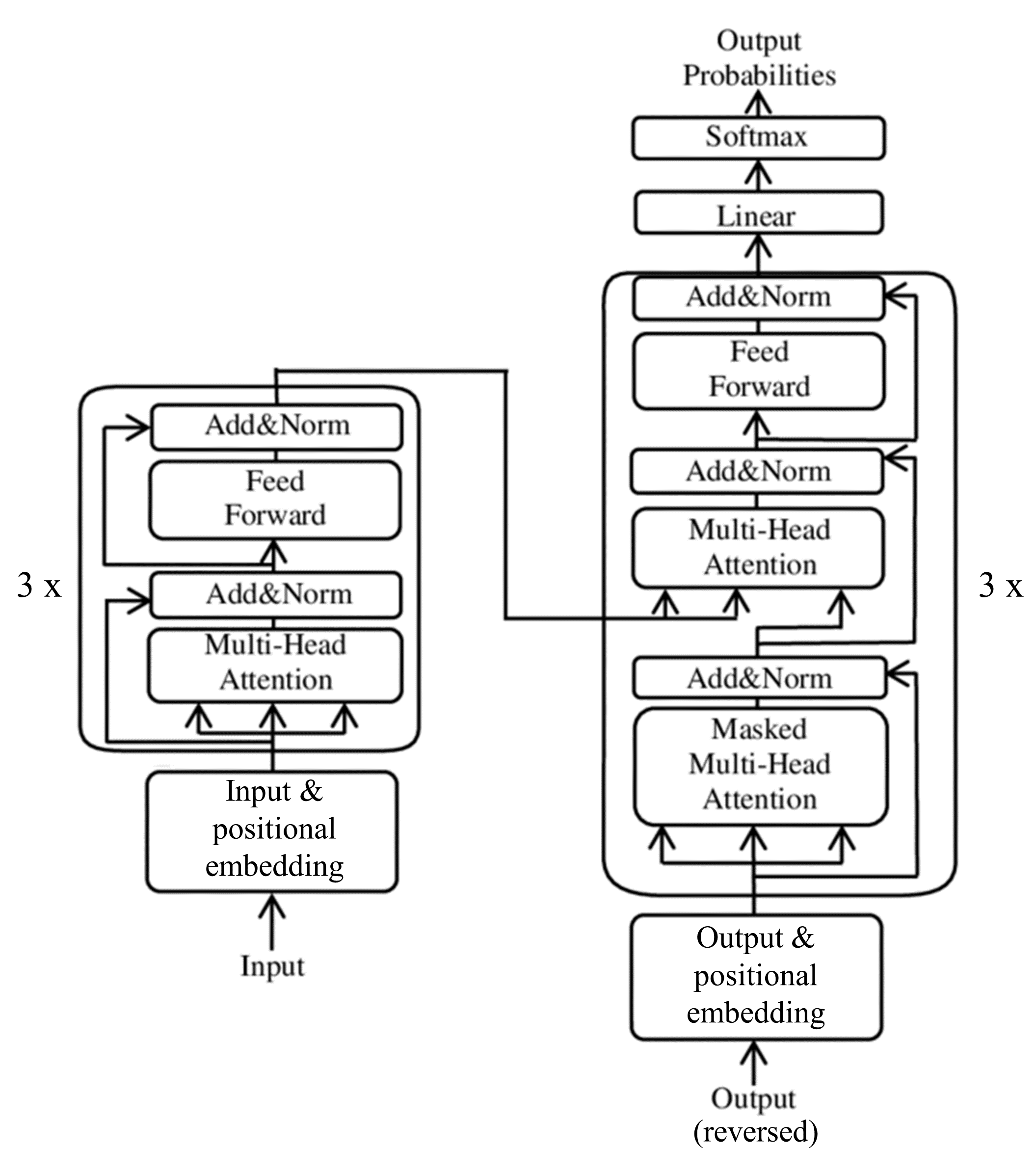


Additional file 1: Figure S3. Figure of transformer architecture, adapted from a work by Yuening Jia from DOI:10.1088/1742-6596/1314/1/012186 under CC BY-SA 3.0.

Distribution of the types of errors that occur in the invalid sequences generated by the *de novo* generators (total counts).


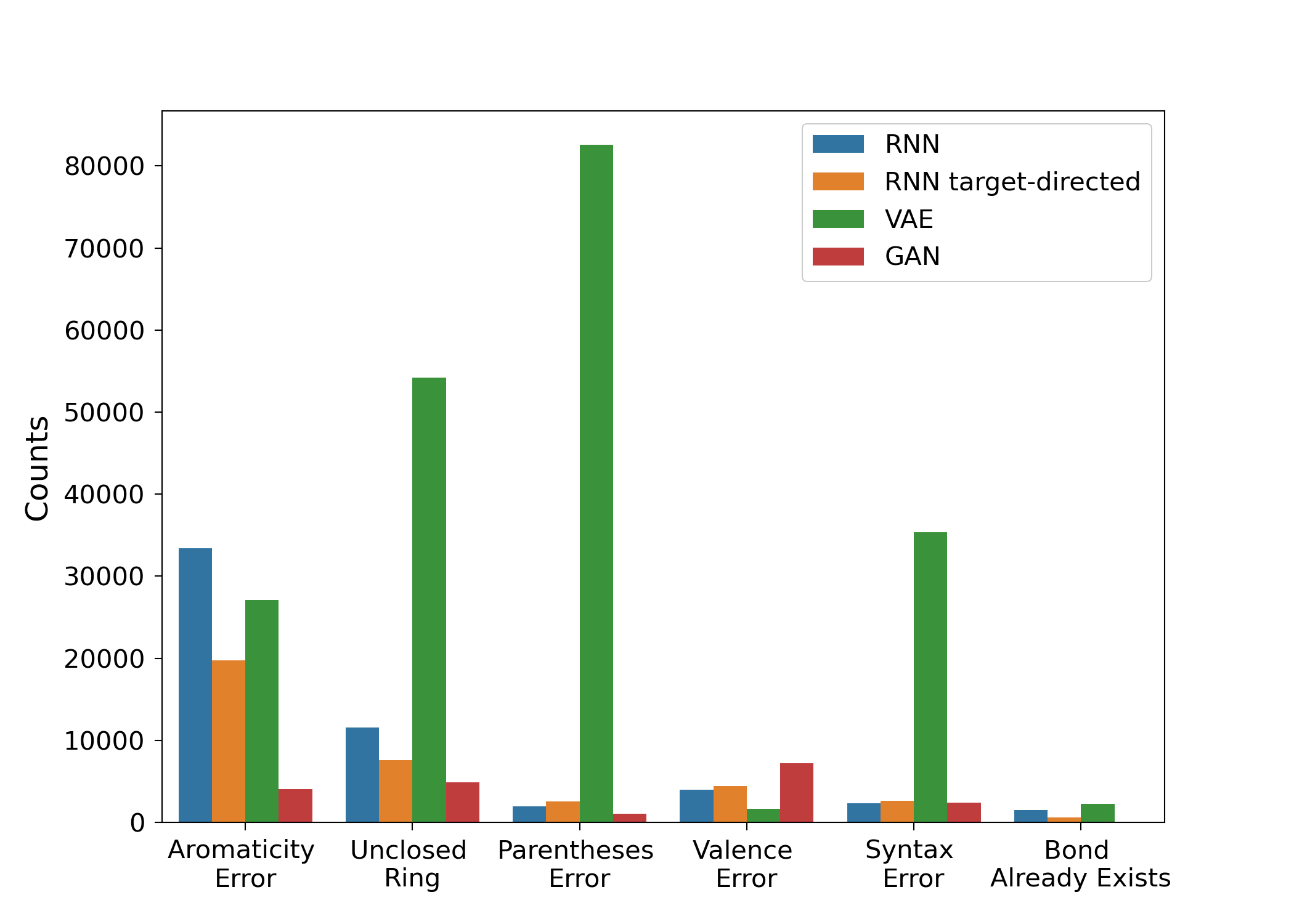


Additional file 1: Figure S4. The errors that are present in the synthetic datasets based on the papyrus dataset.

Plots of errors from synthetic datasets as categorized by the RDKit


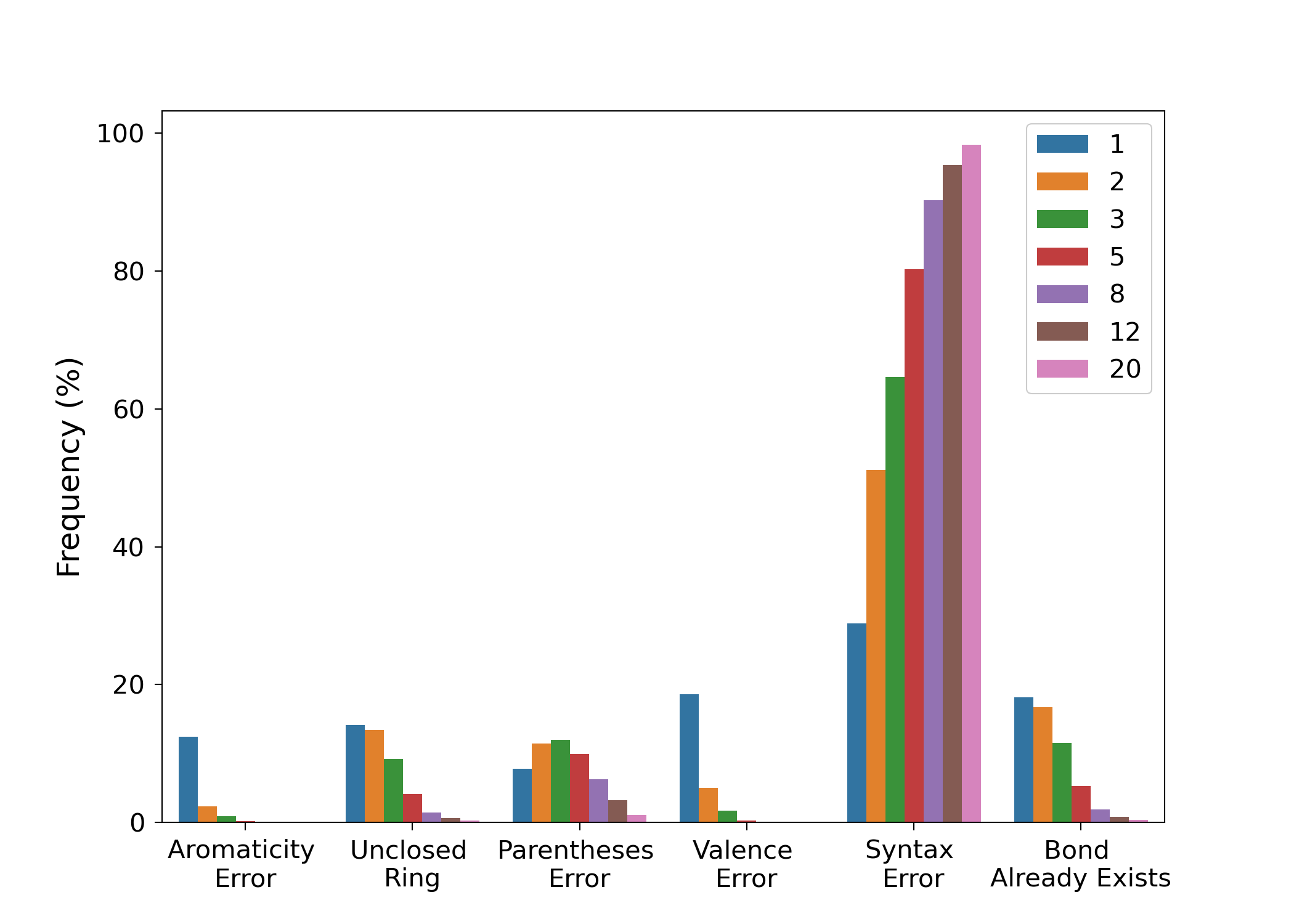


Additional file 1: Figure S5. The errors that are present in the synthetic datasets based on the papyrus dataset.

KL divergence

**
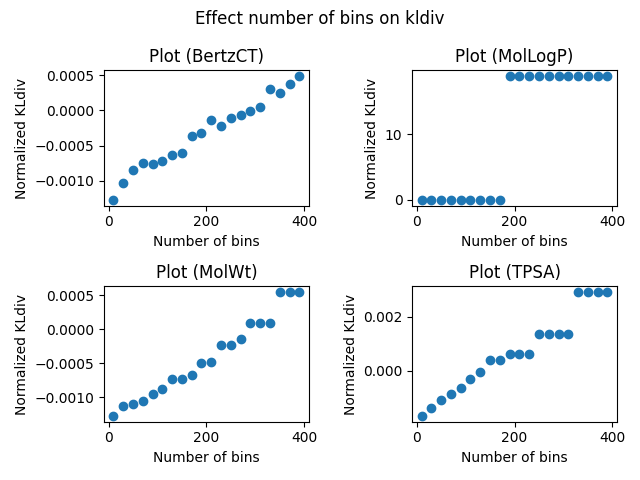
** Additional file 1: Figure S6. Effect of number of bins on KL divergence score. The plots show the divergence score of smoothened histograms with different numbers of bins compared to the divergence score based on kernel density estimation.

Original values table 1

Additional file 1: Table S2. Comparison of fixed, generated, and training set molecules for the 3 general generative model case studies is given. Uniqueness is the fraction of unique molecules in a sample of 10,000 valid molecules. Novelty is the fraction of 10,000 molecules that are not present in a sample of 100,000 from the reference set. For the similarity metrics, 10,000 molecules were compared to 100,000 molecules from the reference set. SNN is the similarity to the nearest neighbor. Fragment and scaffold similarity are calculated by comparing the frequency distribution of different fragments or scaffolds compared to the reference set. KL divergence describes the similarity of the physiochemical property distributions of 10,000 molecules compared to the reference set.

| Case | Case – reference | Uniqueness^a^ | Novelty | Similarity | | | KL divergence |
| --- | --- | --- | --- | --- | --- | --- | --- |
|  |  |  |  | SNN | Fragment | Scaffold |  |
| RNN | Fixed – generated | 1.00 | 1.00 | 0.42 | 1.00 | 0.26 | 0.94 |
|  | Fixed – train |  | 1.00 | 0.42 | 1.00 | 0.25 | 0.96 |
|  | Generated – train | 1.00 | 1.00 | 0.51 | 1.00 | 0.55 | 0.99 |
| VAE | Fixed – generated | 1.00 | 1.00 | 0.38 | 0.98 | 0.37 | 0.80 |
|  | Fixed – train |  | 1.00 | 0.39 | 0.99 | 0.30 | 0.96 |
|  | Generated – train | 1.00 | 1.00 | 0.37 | 0.97 | 0.43 | 0.90 |
| GAN | Fixed – generated | 0.92 | 1.00 | 0.55 | 0.82 | 0.84 | 0.58 |
|  | Fixed – train |  | 1.00 | 0.42 | 0.79 | 0.36 | 0.48 |
|  | Generated – train | 1.00 | 1.00 | 0.31 | 0.68 | 0.21 | 0.37 |

^a^Uniqueness is calculated for the fixed and generated set

Performance QSAR models

**
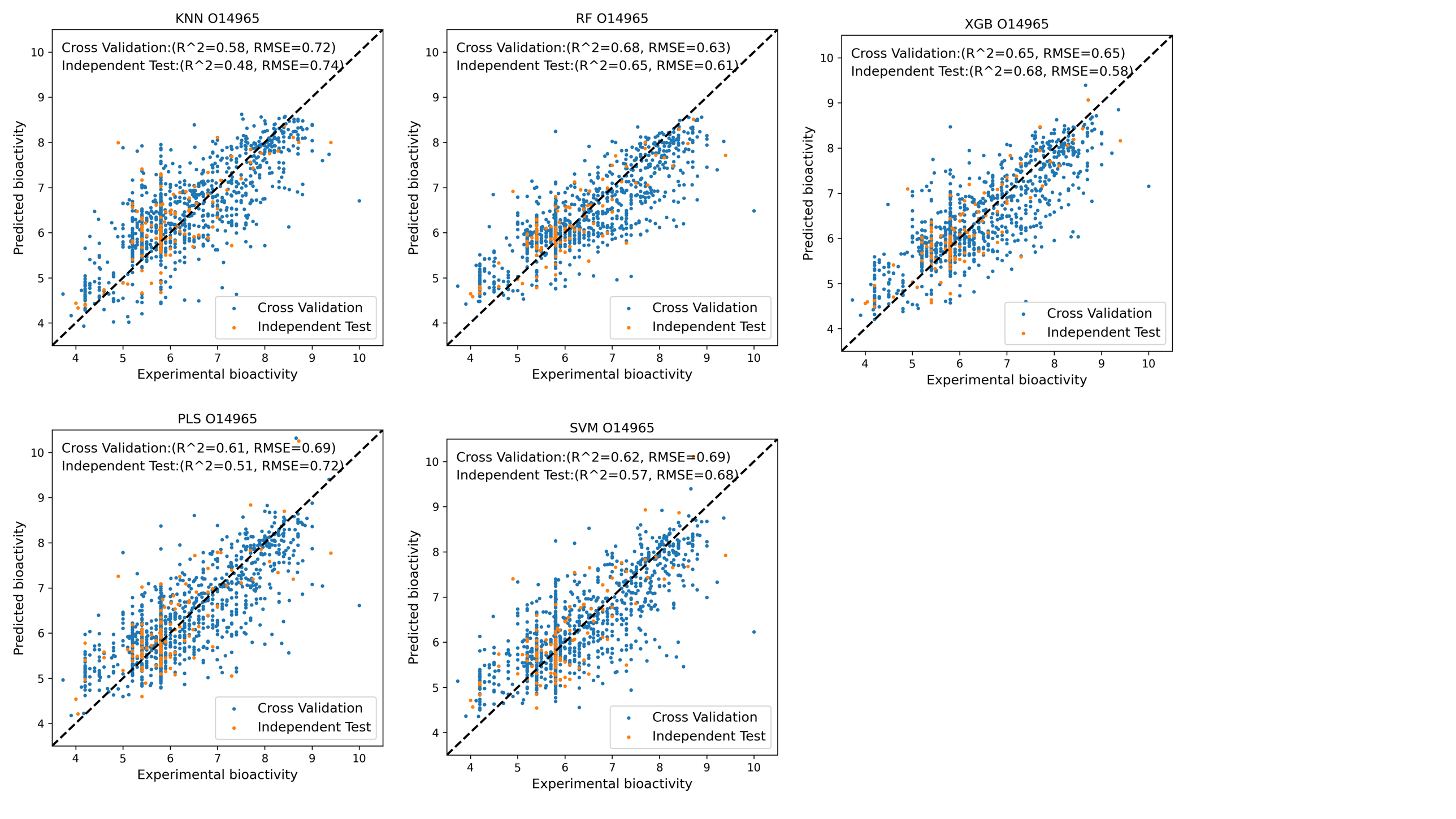
** Additional file 1: Figure S7. Performance of QSAR models for Aurora kinase A.

**
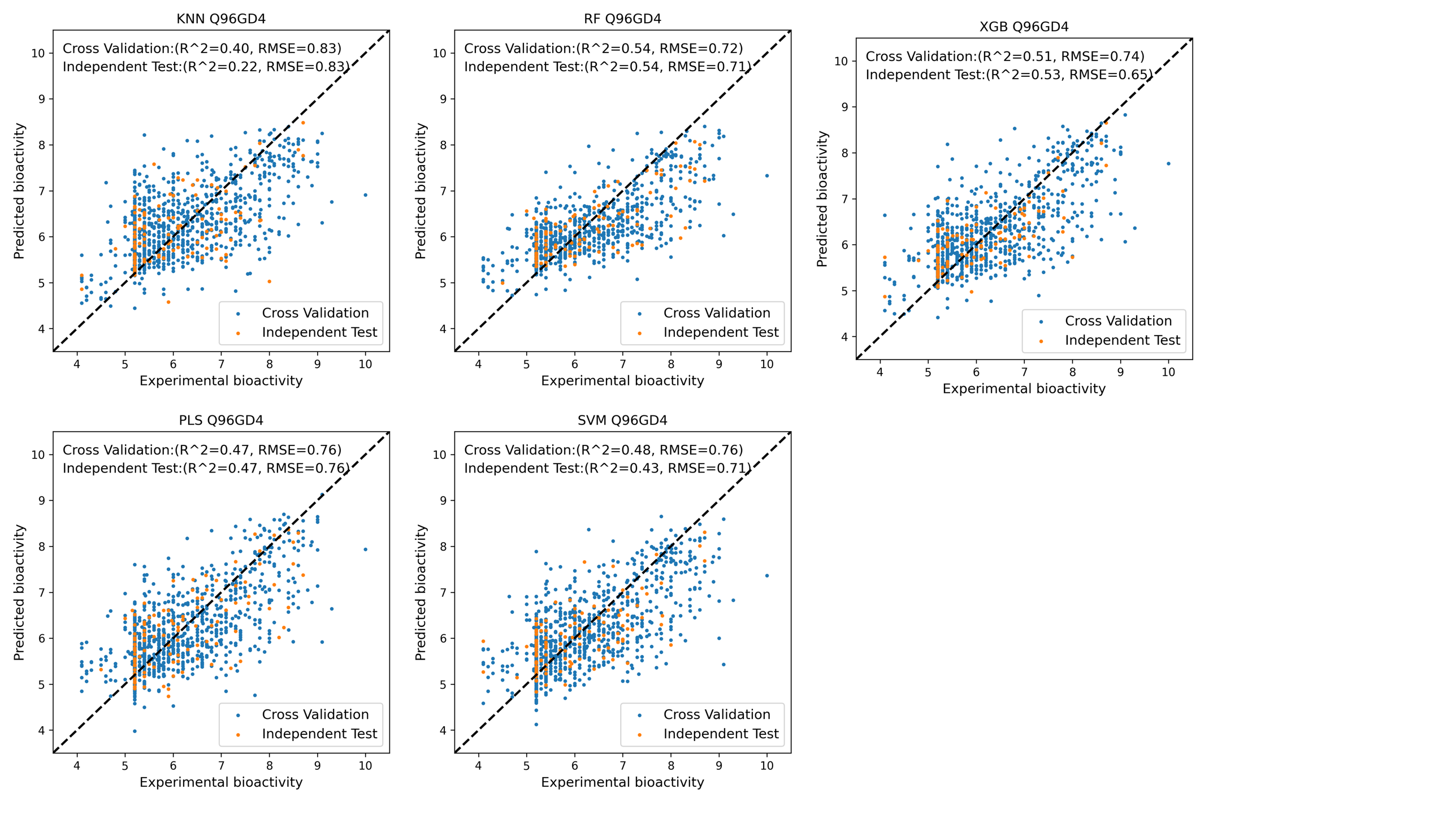
** Additional file 1: Figure S8. Performance of QSAR models for Aurora kinase B.

**
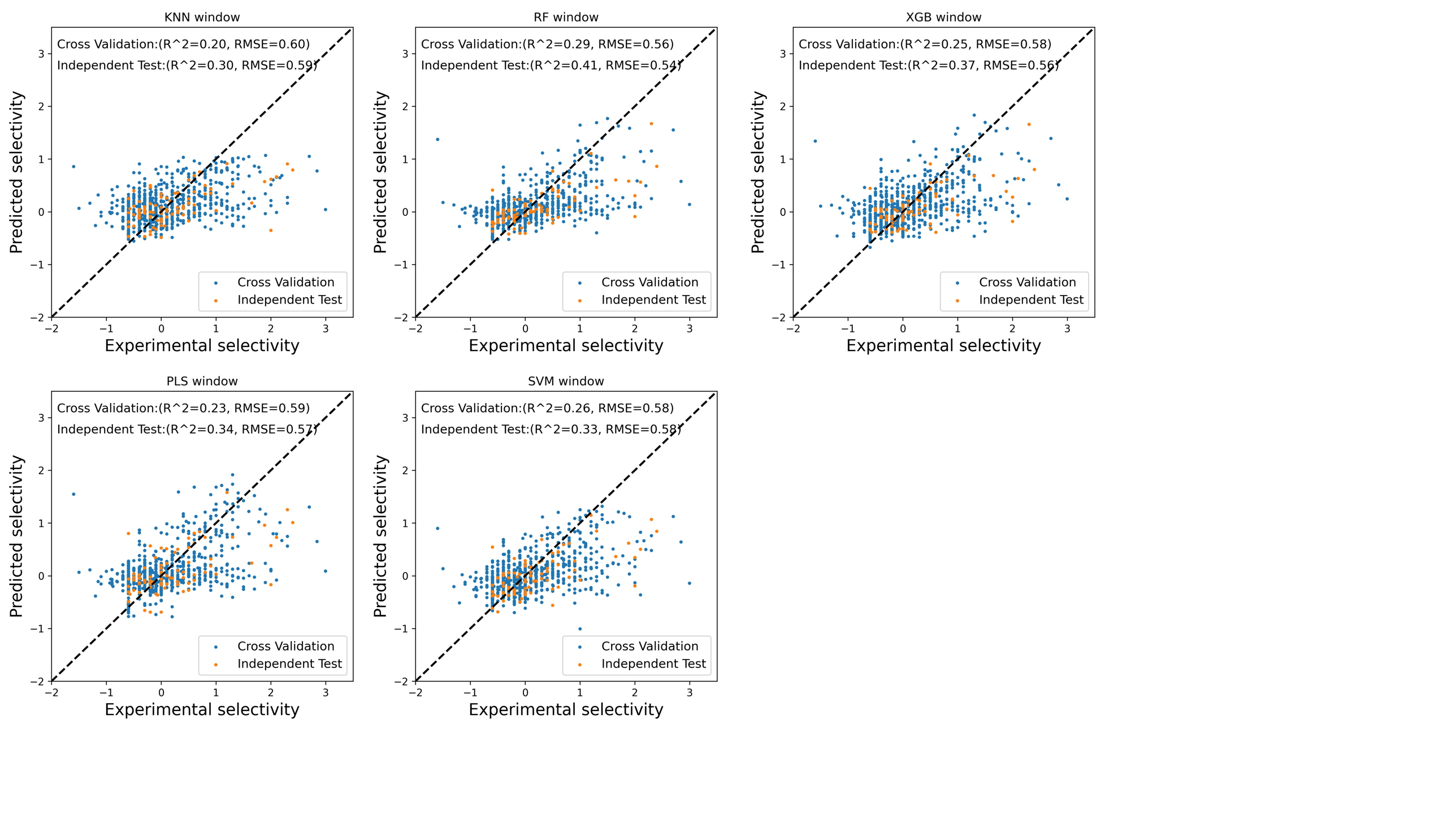
** Additional file 1: Figure S9. Performance of selectivity window QSAR models.
